# Comparison of Postoperative Complications of Open Versus Laparoscopic Cholecystectomy According to the Modified Clavien-Dindo Classification System

**DOI:** 10.7759/cureus.43642

**Published:** 2023-08-17

**Authors:** Aqsa Khalid, Kalsoom Khalil, Haseeb Mehmood Qadri, Chaudhary Zeeshan Ahmad, Warda Fatima, Ali Raza, Muhammad Ahsan Asif, Muhammad Shaheer Luqman, Jawariah, Muhammad Faraz K Nizami

**Affiliations:** 1 Surgery, Lahore General Hospital, Lahore, PAK; 2 Surgery, Shaikh Zayed Hospital, Lahore, PAK; 3 Surgery, Jinnah Hospital, Lahore, PAK; 4 Surgery, Independent University Hospital, Faisalabad, PAK

**Keywords:** pakistan, gall bladder, surgery, grade, postoperative complications, modified clavien dindo classification, cholecystectomy

## Abstract

Background: Though laparoscopic cholecystectomy has become a gold standard management technique for gallbladder diseases, an open approach can also be used for patients having complicated gallbladder disease. Post-cholecystectomy complications are well-documented in existing English scientific literature but are not well understood according to the grade of intervention required to treat those complications.

Objective: To compare the postoperative complications of laparoscopic versus open cholecystectomy according to the modified Clavien-Dindo classification (MCDC) system.

Materials and methods: A retrospective study was conducted at the Department of General Surgery, Unit - III, Lahore General Hospital, Lahore, comprising the data of patients operated between July 01, 2021, and December 31, 2021, after departmental approval # SU-III/73/LGH, dated April 1, 2022. Patients with the definitive diagnosis of acute cholecystitis, chronic cholecystitis, cholelithiasis, and cholecysto-duodenal fistula were included, while cases of choledocholithiasis and, gall bladder carcinoma were excluded from this study. Eighty patients met the inclusion criteria, with 40 patients in each group of open and laparoscopic cholecystectomy. Information for the data set of age, gender, history of surgical procedure, immediate and late outcome, length of surgery, and MCDC grade were collected. Low-grade complications were Grade I and Grade II, while Grades III to V were high-grade.

Results: The mean age of included patients was 42.52 ± 8.76 and 40.025 ± 8.12 years, in the open and laparoscopic group, with 80% and 90% female preponderance, respectively. Grade I and Grade II complications occurred in both groups of patients, with Grade III only in patients who underwent open cholecystectomy. None of the patients from each group developed Grade IV or Grade V complications. Among 40 patients who underwent laparoscopic cholecystectomy, 35% of the patients developed low-grade complications, whereas 40% of the patients developed low-grade complications after open cholecystectomy, with respiratory complications being the most common. High-grade complications after open cholecystectomy were found among 2.5% of patients, whereas no patients developed high-grade complications following the laparoscopic approach.

Conclusion: Patients who underwent laparoscopic cholecystectomy are less prone to develop complications than patients undergoing open cholecystectomy, hence requiring low-grade interventions of surgical and non-surgical types. MCDC is a valuable tool for assessing surgical complications and can help improve patient outcomes by providing a standardized method for reporting and comparing complication rates.

## Introduction

Gallstone disease has been a prime cause of morbidity around the globe. In Pakistan, the disease prevalence has been reported to be 10% [1]. The laparoscopic cholecystectomy is the gold standard of therapy for acute cholecystitis and chronic cholecystitis [2]. However, patients who suffer from complicated gall bladder pathologies, for example, difficult anatomy, extensive inflammation, intra-abdominal adhesions, gall bladder cancer, and inexperience to handle equipment require an open surgical approach [3]. The laparoscopic procedure is preferred over the open approach owing to its benefits of decreased pain, shorter convalescence, reduced intra-operative stress, and limited inflammatory response [4]. According to a study in the World Journal of Gastroenterology by Antoniou et al., the mortality rate associated with the laparoscopic approach is one percent and 4.4% with the open approach; cardiac complications emerged in 0.6% of the patients treated via the laparoscopic approach and in 1.2% of the patients treated via open surgery [4]. The commonly reported immediate complications are infection, hemorrhage, and injury to the bile duct, bowel wall, and blood vessels. The early postoperative complications are postoperative hemorrhage, abscess, and retained stones. The late postoperative complications are choledocholithiasis, biliary stenosis, and remnant gall bladder [5,6]. Overall, the most common complications consist of retained stones (12%), wound infection (10%), and bile leakage (9%) [6]. Coccolini et al. document in their meta-analysis that the laparoscopic approach reduces hospital stay and postoperative complications; however, it has no effect on postoperative complications, such as bile leakage and severe hemorrhage [2].

There is a scarcity of existing English scientific literature comparing open versus laparoscopic cholecystectomy, according to the modified Clavien-Dindo classification (MCDC) system. To the best of our knowledge, after the literature search from PubMed, Google Scholar, and Scopus, we conclude that this is the first study from Pakistan comparing the postoperative complications of open versus laparoscopic cholecystectomy (OC vs. LC), using the MCDC system.

## Materials and methods

This retrospective, observational study was conducted after departmental approval # SU-III/73/LGH, dated April 1, 2022, at the Department of General Surgery, Unit - III, Lahore General Hospital, in the month of May 2022. Patients who underwent cholecystectomy between July 1, 2021, and December 31, 2021, were enrolled in this study.

Inclusion criteria

The inclusion criteria were as follows: 1) all patients of age 18 years or more, irrespective of their gender; 2) patients with the definitive diagnosis of acute cholecystitis, chronic cholecystitis, cholelithiasis, and cholecysto-duodenal fistula were included.

Exclusion criteria

The exclusion criteria were as follows: 1) cases of choledocholithiasis, gall bladder carcinoma, and cholangiocarcinoma; 2) pediatric population less than 18 years of age; 3) cases with incomplete or missing records.

Eighty patients met the criteria, with 40 patients in each group of open and laparoscopic cholecystectomy. Information for the data set of age, gender, history of surgical procedure, immediate and late outcome, length of surgery, and MCDC grade were collected via Google Forms. This information was transferred to IBM SPSS Statistics for Windows, Version 26.0 (IBM Corp., Armonk, NY) and sent to a statistician for descriptive analysis. The MCDC system (Table 1) was used in our study to compare the postoperative complications of open and laparoscopic cholecystectomy [5].

**Table 1:** Modified Clavien-Dindo classification (MDC) system

Low-grade complications	
Grade I	Any deviation from the normal intraoperative or postoperative course, including the need for pharmacologic treatment other than antiemetics, antipyretics, analgesics, diuretics, electrolytes, or physiotherapy
Grade II	Complications needing only the use of intravenous medications, total intravenous nutrition, or blood transfusion
High-grade complications	
Grade Ill (a)	Complications needing surgical, endoscopic, or radiologic intervention under local anesthesia
Grade Ill (b)	Complications needing surgical, endoscopic, or radiologic intervention under general anesthesia
Grade IV (a)	Life-threatening complications requiring ICU management - single organ dysfunction (including hemodialysis)
Grade IV (b)	Life-threatening complications requiring ICU management - multi-organ dysfunction
Grade V	Death of the patient

Operational definitions

Immediate outcome was defined as the occurrence of any complication, referral, or discharge within 24 hours of surgery. Late outcome was defined as the occurrence of any complication, referral, or discharge after 24 hours of surgery.

## Results

The cases of open cholecystectomy included 20% male and 80% female patients, and their mean age was 42.52 ± 8.76 years. Among the patients, 10% were residents of rural areas and 90% of urban areas. On the other hand, gender distribution in the cases of laparoscopic cholecystectomy was 10% male and 90% female patients, respectively. Their mean age was 40.025 ± 8.12 years and they were all (100%) from urban areas (Table 2).

**Table 2:** Approach-specific comparison HDU: high dependency unit

Sr. #	Parameters	Open cholecystectomy	Laparoscopic cholecystectomy
1.	Average hospital stay (days)	9.55 days	7.2 days
2.	Common indications		
	Cholelithiasis	87.5%	92.5%
	Acute cholecystitis	7.5%	2.5%
	Chronic cholecystitis	2.5%	5%
	Fistula	2.5%	Nil
3.	Positive history of previous surgery	42.5%	55%
4.	Surgical procedure duration (hours)		
	< 1 hour	2.5%	25%
	1 – 2 hours	57.5%	67.5%
	2 – 3 hours	37.5%	7.5%
	>3 hours	2.5%	
5.	Immediate patient outcome	Shifted to the ward within an hour: 100%	Shifted to the ward within an hour: 97.5%, shifted to HDU: 2.5%
6.	Late patient outcome	Recovery from complications and discharge: 100%	Recovery from complications and discharge: 100%

We have summarized the postoperative complications for both approaches of cholecystectomy in Table 3. Grade I and Grade II complications occurred in both groups of patients, with Grade III only in patients who underwent open cholecystectomy. None of the patients from each group developed Grade IV or Grade V complications.

**Table 3:** Comparison of postoperative complications using the modified Clavien-Dindo classification (MCDC) system

Sr. #	Parameters	Open cholecystectomy	Laparoscopic cholecystectomy
1.	Occurrence of type of complications postoperatively		
	Low-grade	40%	35%
	High-grade	2.5%	Nil
	Both	Nil	Nil
	None	57.5%	65%
2.	Type of low-grade complication(s)		
	Grade I	37.5%	35%
	Grade II	2.5%	Nil
	Both	2.5%	Nil
	None	57.5%	65%
3.	Most common grade I complication(s)		
	Respiratory complaints	15%	15%
	Fever	12.5%	10%
	Nausea and vomiting	10%	12.5%
4.	Most common grade II complication(s)		
	Bleeding	4%	Nil
5.	Type of high-grade complication(s)		
	Grade III	2.5%	Nil
	Grade IV	Nil	Nil
	Grade V	Nil	Nil
	All	Nil	Nil
	None	Nil	Nil
6.	Type of grade III complication(s)		
	Grade IIIa	2.5%	Nil
	Grade IIIb	Nil	Nil
7.	Most common grade III complication(s)	Surgical site infection requiring incision and drainage under local anesthesia	Nil
8.	Grade IV complication(s)	Nil	Nil
9.	Grade V complication(s)	Nil	Nil
10.	Final patient outcome	Recovered from complications and discharged: 100%	Recovered from complications and discharged: 100%

## Discussion

There is an abundance of existing English scientific literature on post-cholecystectomy complications and their management. But, the need and degree of interventions required to manage those complications are missing. This study is conducted in a tertiary care set-up where we retrospectively assessed the postoperative complications of patients undergoing laparoscopic and open cholecystectomy and graded those complications according to the degree of non-surgical or surgical interventions required to manage them.

The affected patients in both groups at our center were in their fifth decade, with at least four-fifths female predominance in each group. In previous studies, 80% of patients were younger than 70 years, with 63% of females undergoing LC and 51% of males undergoing OC [7]. Cholelithiasis and acute cholecystitis were the two most frequent indications of surgical intervention at our center. This finding is consistent with another study done by Sapmaz et al., in which the most common diagnosis made was cholelithiasis followed by acute cholecystitis [8]. However, in a Pakistani study, chronic calculus cholecystitis was the most common indication [9]. A recent Pakistani study describing the complications of laparoscopic cholecystectomy by Amreek et al. demonstrates a high prevalence rate of 10% in Pakistan [1]. This high incidence may be explainable by the sedentary lifestyle and obesity epidemic in this country.

Of the total, 67.5% and 57.5% of patients had been operated on between 1-2 hours for OC and LC, respectively. This percentage is lower than the one highlighted by Taki-Eldin and Badaway, where 95.1% of patients who were managed with LC were operated on in <1.5 hours [10]. This difference can again be explained on the basis of our smaller sample size. All the patients after OC were shifted to the ward within an hour, while 97.5% of patients after LC were shifted to the ward within an hour, with the remaining 2.5% admitted to the high dependency unit (HDU). These factors depict that LC was superior to OC, in terms of duration of operation, but inferior to OC in terms of immediate postoperative recovery period.

Analysis of data from all the patients who underwent open and laparoscopic cholecystectomy showed different complications. We graded these complications according to the MCDC system. In our study, 40% of the patients with OC and 35% of the patients with LC developed low-grade complications. The most common type of grade 1 complication was a respiratory complaint, i.e. decreased bibasilar breathing due to intra-operative atelectasis in both cases, followed by a fever that was more common in patients with OC. Bleeding was the most common grade 2 complication occurring only in OC cases with none reported in patients with LC. Owing to a limitation in sample size, it is in contrast with the findings of Zaidi and colleagues in 2015 who showed that out of 1500 patients, 1.33% cases reported per operative hemorrhage, i.e. 20 out of 1500 patients [11]. Among grade 3 complications, surgical site infection was the commonest, occurring in those who had undergone OC with zero occurrence in cases with LC. We identified no grade 4 and grade 5 problems in our study owing to the proper surgical handling, in contrast to other studies where mortality was 1.0% with laparoscopic cholecystectomy and 4.4% with open cholecystectomy [4].

The average hospital stay in our study is 9.55 days for OC and 7.2 days for LC. Increased hospital stay in our study was mainly due to grade 1 complications. However, the fact that the average stay for patients with LC is less than those with OC is supported by a meta-analysis that reported the mean stay to be 4.1 days less in cases of LC than in patients who had undergone OC [2]. Previous surgical history was present in 42.5% and 55% of OC and LC patients respectively, whereas a previous study found this history only in 1.7% and 0.3% cases, respectively [7]. This huge difference can be explained by the fact that the study mentioned later had a huge sample size and thus its results can be generalized to a large group of population.

There is ample data available comparing outcomes of OC versus LC. This study is unique since there are no studies from Pakistan that perform this comparison on the basis of the MCDC system.

Limitations

The authors endorse the limitations of the study. The fact that it is a single-center audit used to assess the quality, efficiency, and effectiveness of procedures at a specific location with a small sample size is a limiting factor; although we will consider enhancement in the sample size in the future to further explain the importance of this topic. The scarcity of existing literature on the same topic still is another reason why constructive comparisons could not be made. Due to the small sample size, a productive comparison could not be made. We will prioritize enhancement in the sample size in the future to further explain the significance of this topic. A specific number of people can also be confined to a specific topic in the future for any comparison.

Clinical recommendations

The MCDC system provides a standardized method for assessing and reporting surgical complications, which can help to improve patient outcomes. To implement the MCDC system in our surgical practice, we should train our surgeons and other healthcare workers on how to use the system to assess and report surgical complications. This could involve incorporating the MCDC system into our existing surgical protocols and file records.

## Conclusions

Our study has found that laparoscopic cholecystectomy procedures are not only safer than traditional open cholecystectomy for patients, but they also offer other benefits such as shorter hospital stays and lower costs for healthcare providers. These advantages make laparoscopic procedures an attractive option for patients and healthcare providers alike. We came to this conclusion after conducting a retrospective analysis of patient outcomes using the modified Clavien-Dindo classification (MCDC) system. The MCDC system is a valuable tool for assessing surgical complications and can help improve patient outcomes by providing a standardized method for reporting and comparing the rates of complications.
